# A Computational Study on Selected Alkaloids as SARS-CoV-2 Inhibitors: PASS Prediction, Molecular Docking, ADMET Analysis, DFT, and Molecular Dynamics Simulations

**DOI:** 10.1155/2023/9975275

**Published:** 2023-05-03

**Authors:** Md. Golam Mortuza, Md Abul Hasan Roni, Ajoy Kumer, Suvro Biswas, Md. Abu Saleh, Shirmin Islam, Samia Sadaf, Fahmida Akther

**Affiliations:** ^1^Department of Pharmaceutical Sciences, North South University, Dhaka 1217, Bangladesh; ^2^Department of Science and Humanities, Bangladesh Army International University of Science and Technology, Cumilla 3500, Bangladesh; ^3^Department of Chemistry, European University of Bangladesh-EUB, Dhaka 1216, Bangladesh; ^4^Miocrobiology Laboratory, Department of Genetic Engineering and Biotechnology, University of Rajshahi, Rajshahi 6205, Bangladesh; ^5^Department of Genetic Engineering and Biotechnology, University of Chittagong, Chittagong 4331, Bangladesh; ^6^Department of Pharmacy, University of Chittagong, Chittagong 4331, Bangladesh

## Abstract

Despite treatments and vaccinations, it remains difficult to develop naturally occurring COVID-19 inhibitors. Here, our main objective is to find potential lead compounds from the retrieved alkaloids with antiviral and other biological properties that selectively target the main SARS-CoV-2 protease (*M*^pro^), which is required for viral replication. In this work, 252 alkaloids were aligned using Lipinski's rule of five and their antiviral activity was then assessed. The prediction of activity spectrum of substances (PASS) data was used to confirm the antiviral activities of 112 alkaloids. Finally, 50 alkaloids were docked with *M*^pro^. Furthermore, assessments of molecular electrostatic potential surface (MEPS), density functional theory (DFT), and absorption, distribution, metabolism, excretion, and toxicity (ADMET) were performed, and a few of them appeared to have potential as candidates for oral administration. Molecular dynamics simulations (MDS) with a time step of up to 100 ns were used to confirm that the three docked complexes were more stable. It was found that the most prevalent and active binding sites that limit *M*^pro^'sactivity are PHE294, ARG298, and GLN110. All retrieved data were compared to conventional antivirals, fumarostelline, strychnidin-10-one (L-1), 2,3-dimethoxy-brucin (L-7), and alkaloid ND-305B (L-16) and were proposed as enhanced SARS-CoV-2 inhibitors. Finally, with additional clinical or necessary study, it may be able to use these indicated natural alkaloids or their analogs as potential therapeutic candidates.

## 1. Introduction

SARS-CoV-2 affects the respiratory system, central nervous system, liver, heart, and kidneys, causing organ failure. Coughing, exhaustion, a loss of taste and smell, discomfort, and sore throat are typical symptoms. From these symptoms, approximately 10% of individuals can develop a severe illness requiring hospitalization and oxygen [1, 2]. Till today, no specific antiviral or SARS-CoV-2 inhibiting medication exists to treat this life-threatening illness. Antiviral medicines, such as lopinavir [3], danoprevir [4], ritonavir [5, 6], remdesivir, umifenovir [7], chloroquine, and hydroxychloroquine [8], as well as antipyretics and mechanical respiratory support [9], are some therapeutic choices. The current pharmacological recommendations for treating coronavirus disease in 2019 (COVID-19) are based on the results of a number of studies that looked at severe acute respiratory problems. Western medicine works better when combined with herbal remedies and/or natural components from medicinal plants than when used alone [10–12]. Two proteases—a 3-C-like protease (*M*^pro^) and a papain-like protease (PLpro)—that are involved in the processing and releasing of translated nonstructural proteins (NSPs) are encoded by the coronavirus polyprotein [13, 14]. Main protease (*M*^pro^), which is essential for COVID-19 replication and maturation, has been identified as a therapeutic target among coronaviruses. SARS-CoV-2, a new coronavirus infection, is caused by an organism with a 30 kB single-stranded RNA genome [15]. The weight of the full molecular structure of this protein (PDB ID: 6M03), which has 306 amino acid residues and deposited residues, 2454 atoms in all, is 33.83 kDa [16]. The search for natural molecules to reduce COVID-19 remains a challenging problem despite the creation of various vaccines and supplementary therapies. To suppress SARS-CoV-2 *M*^pro^, a number of inhibitors have been proposed, including cilexitil, chloroquine, hydroxychloroquine, atazanavir, disulfiram, dipyridamole, candesartan sulfacetamide, cimetidine, and maribavir [17]. Natural substances and alkaloid analogs have recently been the subject of intensive research for their robust antioxidant, antibacterial, anti-inflammatory, and wide antiviral effects [18, 19]. Natural substances may have antiviral capabilities, according to certain recent studies, and they may even be essential in the fight against COVID-19 [20–24]. Furthermore, research on SARS-CoV-2 inhibitory lead compounds derived from bioactive alkaloids is extremely scarce [20, 25]. Alkaloids are typically found in plants, natural foods, fruits, and vegetables. They are nitrogen-containing molecules with at least one nitrogen acting as a heteroatom. Alkaloids are, therefore, likely to contain SARS-CoV-2 antiviral drugs due to their recognized bioactive and antiviral characteristics. Additionally, computational drug design is becoming increasingly important to quickly identify potential drug-like molecules [26, 27]. Lipinski's rule of five was used to pick numerous alkaloids from the PubChem database for this study's purposes, and PASS prediction studies were used to identify compounds with antiviral capabilities. So, the goal of this research is to do an in-depth computational study to find specific lead drugs that can inhabit SARS-CoV-2. In this regard, we studied molecular docking, molecular electrostatic potentials surface (MEPS), molecular dynamics simulation (MDS), and ADMET characteristics of selected alkaloids to find their drug-liking properties as potential lead compounds against the *M*^pro^ of SARS-CoV-2.

## 2. Methodology

### 2.1. Evaluation of Biological Activity

The prediction of activity spectra for substances (PASS) [28] was used to assess the biological activity of the chosen alkaloids. Pa and Pi values were used to forecast whether the ligands were possibly active or inactive. The Pa and Pi values of the proposed active chemical should be near one and zero, respectively. In this work, 256 alkaloids' antiviral capabilities were examined and 50 projected alkaloids with potential antiviral characteristics were taken into consideration for additional research to determine the best SARS-CoV-2 inhibiting drugs.

### 2.2. Protein Preparation

**Figure 1:**
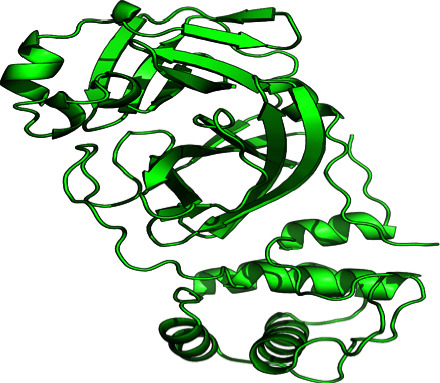
The crystal structure of main protease (*M*^pro^) of SARS-CoV-2 at 2.00 Å resolution (PDB ID: 6M03). Total structure weight: 33.83 kDa, atom count: 2454, modelled residue count: 306, deposited residue count: 306, 3C like unique protein chain.

A single-chain mutation-free protein was chosen from UniPort (https://www.uniprot.org) [29], and the relevant protein was obtained from the RCSB Protein Data Bank (PDB) [30]. Using this, specific proteins in the human body were located. Based on acceptable XRD data, chain number, amino acid residues, resolution, and the lowest value of RMSD, the crystal structure (Figure 1) of the SARS-CoV-2 *M*^pro^ protein (PDB ID: 6M03) was downloaded from the PDB. By eliminating water molecules from the modeled SARS-CoV-2 of *M*^pro^ protein, we prepared it for docking analysis using PyMol version 1.1.0's protein preparation wizard [31] (no cocrystal ligand was attached). Protein energy was minimized using Swiss-Pdb Viewer 4.1.0 [32]. The prepared file was then translated using Open Babel into the PDBQT format [33].

### 2.3. Ligand Preparation

Each alkaloid structure was downloaded in SDF format from the PubChem database site [34]. Then, each alkaloid structure was optimized using the functional B3LYP and basis set 6-31G of the Gaussian 16 program [35].

### 2.4. Molecular Docking

SARS-CoV-2 of *M*^pro^ was the target molecule for docking with the selected ligands, and AutoDock Vina in PyRx, version 0.8, was used for the molecular docking investigation. Vina Wizard predicts the interaction between a protein and a ligand using its scoring function (binding energy in kcal/mol). Based on expected ligand binding sites, the grid box was modified during molecular docking to include all of the binding sites for each protein, and the XYZ coordinates were recorded. The grid center points were set to *X* = 12.11, *Y* = −11.38, and *Z* = 4.66, and the dimension (Å) was set to X = 36.82, Y = 64, and Z = 62.07. Based on the binding energy as well as the probable hydrogen bonds (H-bonds) and hydrophobic contacts, the final protein-ligand interacting models were selected. The calculations of H-bonds and nonbonded interactions were performed using a protein-ligand interaction profiler. The visualization of protein-ligand interaction was carried out using the Discovery Studio, followed by the creation of 3D stereo figures using PyMOL version 1.1.0 [36].

### 2.5. Drug-Likeness Properties

One important factor in the process of finding new drugs is Lipinski's rule of five (RO5). The SwissADME [37] server was used to analyze the Ghose et al. [38], Veber et al. [39], Egan, Muegge rules [38, 40, 41], and bioavailability for the best-interacting ligands among the listed alkaloids.

### 2.6. ADMET Analysis

AdmetSAR [40] and pkCSM-pkinesiotechemkinetics [42] servers were used to know absorption, distribution, metabolism, excretion, toxicity (ADMET), solubility, carcinogenicity, and other pharmacokinetics [43].

### 2.7. Evaluation of Chemical Reactivity by Using Density Functional Theory (DFT)

By analyzing the electrical characteristics of the top 2 bioactive alkaloid ligands, the density functional theory was used to determine the chemical stability of our target molecules. The highest occupied molecular orbital (HOMU) and lowest unoccupied molecular orbital (LUMO) energies are used to calculate global reactivity descriptors. With the help of the following equations, the global reactivity descriptors, such as softness (*S*), electron affinity (*A*), ionization potential 10 (*I*), electronegativity (*χ*), global hardness (*ղ*), global electrophilicity index (*ω*), and chemical potential (*µ*), were determined.

(1) $$\begin{array}{c} E\left(gap\right)=E_{LUMO}-E_{HOMO}\text{,}\\ I=-E_{HOMO}\text{,}\\ A=-E_{LUMO}\text{,}\\ \mu =-\frac{\left(I+A\right)}{2}\text{,}\\ \eta =\frac{\left(I-A\right)}{2}\text{,}\\ S=\frac{1}{\eta }\text{,}\\ \chi =\frac{\left(I+A\right)}{2}\text{,}\\ \omega =\frac{\mu 2}{2\eta }\text{.} \end{array}$$

### 2.8. Molecular Dynamics Simulation

The YASARA dynamics software program and the AMBER14 force field were used to conduct the molecular dynamics simulation of the ligand-protein complexes [44–46]. Initial cleaning, optimization, and orientation of the hydrogen bond network of the docked complexes were performed. The TIP3P solvation model was used to create a cubic simulation cell with periodic boundary conditions [47]. Beyond the complexes of ligand and protein, the simulation cell was elongated by 20 Å each direction. The simulation cell's physiological parameters included pH 7.4, 298 K, and 0.9% NaCl concentration. For the preliminary minimization of energy, the steepest gradient algorithms (5000 cycles) were applied in the simulated annealing method. The time step of the simulation system was adjusted to 1.25 fs. By using the Particle Mesh Ewald (PME) system and a cutoff radius of 8.0 Å, long-range electrostatic interactions were calculated [48]. Simulation trajectory data were saved every 100 ps. Simulations at a constant temperature, pressure, and Berendsen thermostat were conducted for 100 ns. Based on simulation trajectories, the root mean square deviation (RMSD), root mean square fluctuation (RMSF), solvent accessible surface area (SASA), the radius of gyration (Rg), and hydrogen bond were analyzed [44, 48–50].

## 3. Results and Discussion

### 3.1. Estimation of Biological Activities

The PASS online tool was used to evaluate all substances' overall bioactivities based on their chemical structures [51]. The Pa and Pi values of 256 compounds were sorted out with antiviral properties from this server. Only 102 compounds were available with Pa values of more than 0.3 (Table S1). From these data, the first 50 predicted antivirals were used for molecular docking studies against *M*^Pro^ (Table 1 and Table S1). This dataset of predicted antivirals may also be used in any other antiviral drug discovery.

### 3.2. Molecular Docking Analysis

**Figure 2:**
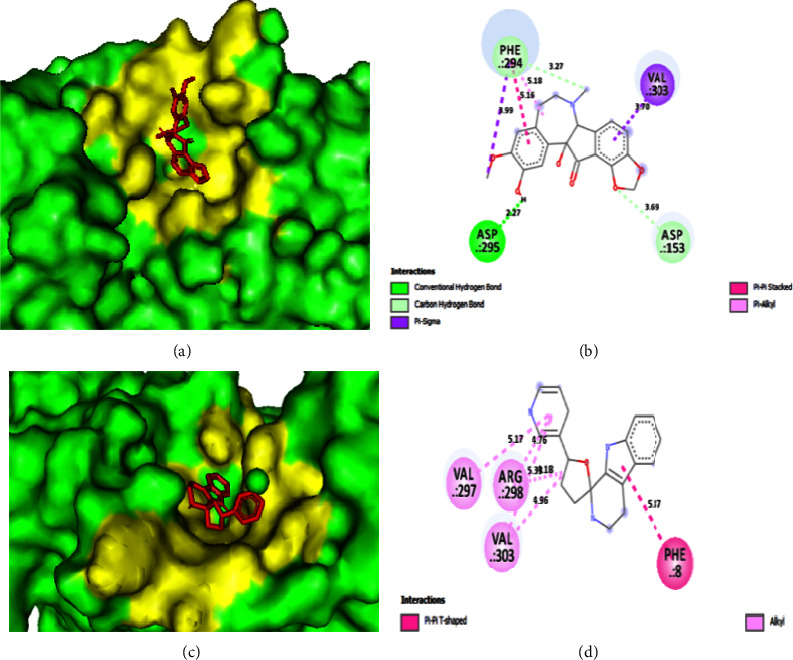
Various poses after docking of selected alkaloids against *M*^Pro^ (6M03). (a and b) 3D and 2D view of *L*-1, respectively; (c and d) 3D and 2D view of *L*-16, respectively.

For determining how well drugs block target receptors, molecular docking is a popular and reliable modeling tool [52]. Out of 50 compounds, fumarostelline and brucine had the highest docking scores, both at −8.5 kcal/mol (*L* − 1 & *L* − 7). In earlier research [53, 54], the alkaloid brucine showed antibacterial and antidengue action. Compared to other licensed antivirals (remdesivir, ritonavir, lopinavir, oseltamivir, and ribavirin), molecules with higher binding energies (remdesivir −7.8 kcal/mol) may be better SARS-CoV-2 inhibitors (Table 2) [55]. The 3D and 2D docking poses of selected L-1 and L-16 alkaloids are shown in Figures 2(a)–2(d), respectively.

### 3.3. Protein Ligand Interaction

Hydrogen bonds and hydrophobic bonds (nonbond interactions) with residues were taken into consideration in the protein-ligand interaction analysis, whereas the others were omitted. Here, in order to determine the bond strength, the bond distance is also computed and taken into account. All relevant data (Table 3) and 2D images of 10 docked interactions with the primary protease (*M*^pro^) of SARS-CoV-2 were recovered. These interactions were *L*-1, *L*-7, *L*-16, *L*-18, *L*-21, *L*-25, *L*-30, *L*-35, *L*-40, and *L*-50 (Table S2). Based on the bond length in hydrogen bonds, practically all ligands (except from *L*-16) are discovered to have strong bonds (2.5–3.1 Å), including the FDA-approved antiviral remdisivir. One of the most prevalent residues for *L*-1, *L*-7, *L*-18, *L*-30, *L*-35, and *L*-40 and remdesivir is PHE294. Similarly, the ligands *L*-7, *L*-16, *L*-18, and *L*-40 all show ARG298 as a binding site. In addition, GLN110 is connected to the drugs remdesivir, *L*-7, *L*-25, and *L*-40. Surprisingly, PHE294, ARG298, and GLN110 demonstrate hydrophobic and hydrogen bonds with the closest bond distances (GLN110: 1.95 Å). Finally, it was found that the most frequent binding sites for a number of chemicals in this *M*^Pro^ were PHE294, ARG298, and GLN110. It has been hypothesized that the formation of the residues PHE294, ARG298, and GLN110 by our suggested lead compounds may limit the function of viral protein *M*^pro^ (6M03).

### 3.4. Molecular and Pharmacokinetic Properties

**Figure 3:**
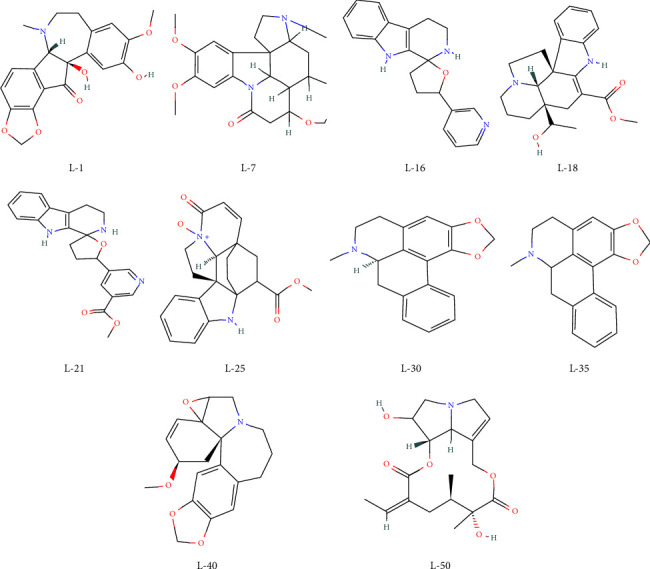
Chemical structures of the top 10 ligands.

The chemical structures of the top ten ligands are shown in Figure 3. Based on molecular analysis of the top ten docking score compounds (Table 4), the number of hydrogen bond acceptors (H. Ac) ranges from 3 to 7 (Lipinski: 10) and the number of hydrogen bond donors (H. Do) ranges from 0 to 2 (Lipinski: 5). For a compound to be a good therapeutic candidate, its H-bond acceptors and donors may not be more than ten and five, respectively [56]. The findings imply that all the shortlisted compounds have the potential to be therapeutic candidates. Topological polar surface area (TPSA) of possible therapeutic candidates ranges from 0 to 140 [57]. In this study, the TPSA values for our short-listed compounds lie between 21 and 88. However, the TPSA values in this instance are 204.0, 148.12, 272.27, 169.93, and 95.44 for the standard antivirals. It is also a matter of consideration or in-depth research to identify *o* find a high standard TPSA value that will work well for approved drugs and potential drug candidates. Lipophilicity (XLOGP3) (Lipinski: XLOGP3 5) was discovered to be upto 3.39 [58]. This indicates that *L*-7, *L*-16, *L*-30, *L*-35, *L*-40, and *L*-50 had a higher affinity for binding to protein, and *L*-1, *L*-18, *L*-2, and *L*-25 had a very low affinity (Log*P*=0 to 1.59) for a lipid environment. This improves drug uptake and metabolism and facilitates the body's ability to absorb the medication through the intestines. Standard ESOL of 4.0 was determined to be within the range of −4.00 to −2.09 for water solubility [59]. More soluble *L*-7 has a higher oral bioavailability and permeability, which suggests that it is better absorbed in the gastrointestinal tract. All of the ten shortlisted lead compounds, which are interestingly all BBB-capable like remdesivir, are safer than ritonavir, lopinavir, oseltamivir, and ribavirin from that perspective. All of the shortlisted compounds, much like the other specified standard medicines, have been found to have acute oral toxicity (AOT) of grade III. Similar to this, all anticipated chemicals have a bioavailability (BA) of around 0.55 and are noncarcinogenic (CAR). These parameters guarantee that the aforementioned lead compounds will be accepted in drug discovery methods.

### 3.5. Drug-Likeliness Properties

Drug research and development processes are sped up through the study of drug-like features. The five guidelines of Lipinski are employed as a criterion for locating possible drug candidates. However, if one rule is broken, it is acceptable to suggest a potential medicine [60]. Table 4 displays the outcomes after molecules are subjected to Lipinski's rule of five, the Ghose filter rule, the Veber rule, the Egan rule, and the Muegge (LGVEM) rule. These criteria have their own rules for determining if a bioactive function is a potent drug. All of the alkaloids chosen for this study precisely followed the LGVEM guidelines without breaking any of them. This demonstrates the discussed chemicals' potential as drugs. Although they are recognized and accepted medications, remdesivir, ritonavir, lopinavir, and ribavirin have been found to have some violations of these LGVEM guidelines. In order to improve the effectiveness of drug-likeliness rules as a component of computational techniques in computer-aided drug design, this also creates a need for a separate, comprehensive study to build a deep association between drug-likeliness rules and parameters of approved medications (CADD).

### 3.6. ADMET Properties of the Selected Compounds

The pharmacokinetics of a drug (also known as absorption, distribution, metabolism, excretion, and toxicity, or ADMET) is the study of how pharmaceuticals enter, travel through, and leave the body [61]. Intestinal absorption in humans is greater than 94% for 9 out of 10 drugs but is only 71% and 65% for remdesivir and lopinavir, respectively. Finally, it showed that, in comparison to some of the traditional medications, the lead compounds indicated above had a higher capability for intestinal absorption by humans (Table 5).

The Caco-2 human colon epithelial cancer cell line permeability assay analyzes the rate of chemical flux through polarized Caco-2 cell monolayers to predict *in vivo* drug absorption. An active or harmful chemical may have an efflux ratio greater than two (https://bienta.net/caco-2-assay/caco-2-validation/). It is interesting to note that all of the suggested lead compounds had Caco-2 permeabilities below 1.75 (0.375 to 1.716). Moreover, *L*-50 had an extremely low permeability (0.375), which was even lower than remdisivir's permeability (0.635). With the exception of *L*-30, *L*-35, and *L*-50, the AMES (Bruce Ames: A test to evaluate chemical carcinogenicity utilizing bacterial strains') toxicity was negative. Based on this investigation, it is concluded that *L*-1, *L*-7, *L*-16, *L*-18, *L*-21, *L*-25, and *L*-40 meet the requirements for oral medications (Table 5).

A drug's bioavailability may be increased by inhibiting P-glycoprotein or decreased by activating P-glycoprotein. When compared to other compounds, the *L*-7, *L*-16, *L*-21, *L*-30, and *L*-35 had a higher capability to block p-glycoprotein, indicating improved drug bioavailability compared to remdesivir, ritonavir, lopinavir, oseltamivir, and ribavirin (Table 5).

### 3.7. Frontier Molecular Orbital of HOMO and LUMO

**Figure 4:**
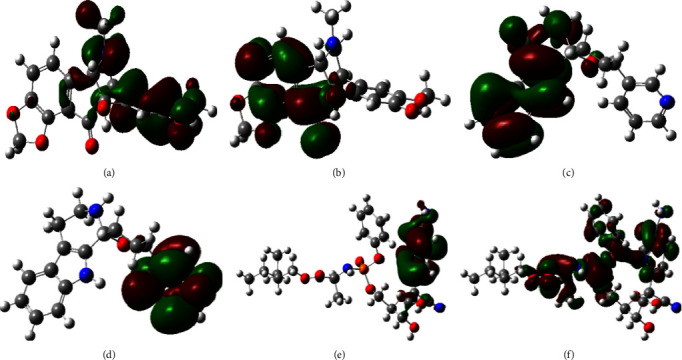
Frontier molecular orbitals diagram for HOMO and LUMO. The HOMO of *L*-1 (a), LUMO of L-1 (b), HOMO of L-16 (c), LUMO of L-16 (d), HOMO of remdesivir (e), and LUMO of remdesivir (f).

Due to their dominance in the coupling of the molecular bridge to electrodes, HOMO and LUMO spatial distributions are typically utilized to describe the transport properties [58]. The chemical reactivity of the top two alkaloids and the HOMO and LUMO frontier molecular orbitals are shown in Table 6 and Figures 4(a)–4(f) in various hues to aid in comprehension. Additionally, a certain color map makes all other molecules accessible and available. Positive nodes are represented by the color green in HOMO, while negative nodes are represented by the deep radish hue. In order to create a chemical bond, the electrophilic attracting group can be linked to the HOMO portion of biologically active molecules, whereas the LUMO is the positive portion that can accept the addition of a nucleophilic group.

### 3.8. Molecular Electrostatic Potentials Surface (MEPS)

**Figure 5:**
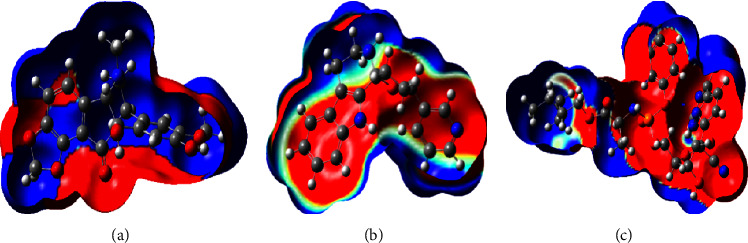
3D map of electrostatic potential charge distribution. (a) *L*-1, (b) *L*-16, and (c) remdesivir.

Figures 5(a)–5(c) illustrate a 3D mapped electrostatic potential charge distribution. This diagram shows the attractive or repulsive force that a fixed charged particle (usually a proton with a point positive charge) experiences at different locations in space that are equally spaced apart from a molecular surface. The negative electrostatic potential (in red) depicts the attraction of the proton by the area of high concentration of electrons in the molecule, whereas the positive electrostatic potential (in blue) corresponds to the repulsion of the proton by atomic nuclei [62, 63].

### 3.9. Molecular Dynamics Simulation

**Figure 6:**
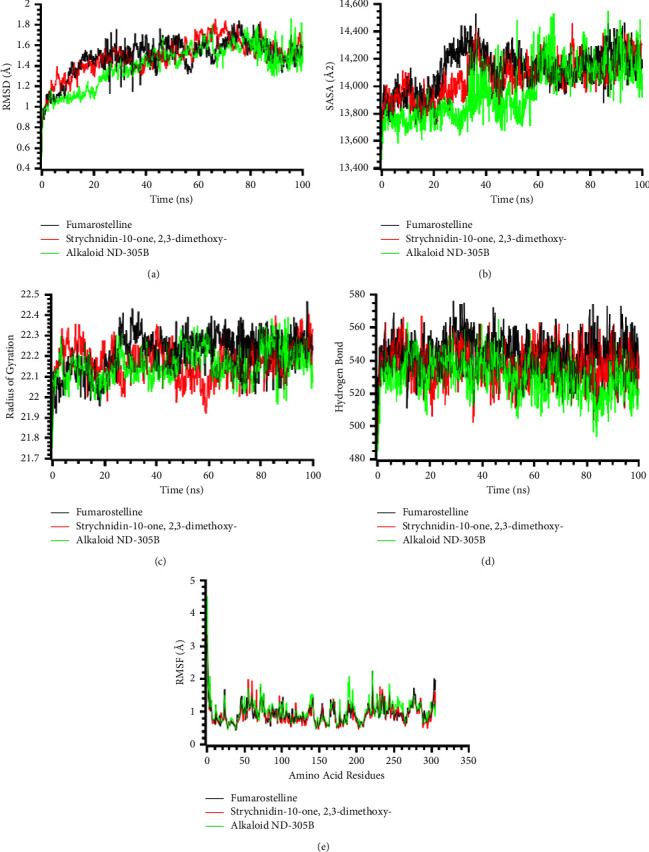
Analysis of all simulated systems on a time-series basis. The RMSD of alpha carbon atoms (a), protein volume with expansion (b), rigidity and compactness analysis (c), hydrogen bonding of the complexes (d), and the flexibility of amino acid residue (e).

To investigate the structural rigidity of the top three protein-ligand complexes and validate the docking scenarios for these complexes, molecular dynamics simulations were performed. The stability of protein-ligand complexes was investigated using the RMSD values of the C-alpha atoms. The ligand-protein complexes containing fumarostelline (*L*-1), strychnidin-10-one, 2,3-dimethoxy (*L*-7), and alkaloid ND-305B (*L*-16) displayed preliminary RMSD increases owing to the instability of these complexes, as shown in Figure 6(a). The fumarostelline-*M*^pro^ complex had a higher increase in RMSD on average than the other two complexes. Contrariwise, the alkaloid ND-305B-*M*^pro^ complex exhibited a lower RMSD value on average than the other two complexes. The RMSD profile of the fumarostelline-*M*^pro^ complex fell drastically at roughly 25 ns, then stabilized at around 45 ns and remained steady with just minor changes for the remaining 55 ns of the simulations. The strychnidin-10-one, 2,3-dimethoxy-*M*^pro^ complex showed a slightly greater RMSD value than the other complexes at 55–70 ns, which could elucidate their enhanced flexibility. Despite this, the RMSD trend of all the three complexes did not surpass 2.5 Å, indicating that the complexes remained stable across the entire simulation time [64].

To measure the variations in the surface of the SARS-CoV-2 main protease (*M*^pro^) in response to interaction with the ligand molecules, the SASA values of the top three docking score complexes were evaluated. The surface area of the protein inflates with the increased value of SASA, while protein truncations occur when SASA decreases [65]. In general, the SASA of the fumarostelline-*M*^pro^ complex was higher on average than the other complexes, suggesting that the complex's surface area was extended than those of the other two (Figure 6(b)). The alkaloid ND-305B-*M*^pro^ complex had lower SASA on average than the other two complexes before peaking at 60 ns of the simulation period. After 60 ns of the simulation time, the fumarostelline-*M*^pro^ complex, strychnidin-10-one, 2,3-dimethoxy-*M*^pro^ complex, and alkaloid ND-305B-*M*^pro^ complex reached a steady-state and remained stable for the remaining 40 ns simulation period with only slight fluctuations. Using Rg values, it was determined whether the protein complexes were more compact or labile. A greater value implies a more labile protein complex, whereas a lower value designates that the simulated protein complex is stiffer. All three complexes displayed an initial increase followed by a decrease of the Rg value. In contrast to the other two complexes, the strychnidin-10-one, 2,3-dimethoxy-M^pro^ complex exhibited relatively low Rg at around 50–70 ns of the simulation period, representing a more stable structure of this complex (Figure 6(c)). Other than that, the other two complexes diverged only slightly but comparatively had lower Rg values.

The docked complexes' hydrogen bonds were examined since hydrogen bonding is important for keeping protein integrity and stability. The fumarostelline-*M*^pro^ complex, strychnidin-10-one, 2,3-dimethoxy-*M*^pro^ complex, and alkaloid ND-305B-*M*^pro^ complex all formed a great deal of hydrogen bonds throughout the simulation trajectory, which signified that the top three ligand molecules formed a tight bond with the *M*^pro^ protein (Figure 6(d)). To further understand *M*^pro^'^s^ flexibility across the amino acid area, the RMSF of the ligand and M^pro^ complexes were investigated. The RMSF profiles of approximately all amino acid residues in the top three docked complexes were below 2.5 Å except at the beginning. The lower RMSF value of the top three docked complexes indicated the decreased flexibility of the complexes since lower RMSF values are connected with the higher stability of the complexes (Figure 6(e)).

## 4. Conclusion

On the basis of computer-aided drug design, which includes protein purification, molecular optimization, molecular docking and visualization, molecular characteristics, ADMET analysis, and molecular dynamic simulations, the first 50 compounds were chosen out of 252 for detailed studies (MDS). The highest possible docking score was −8.5 kcal/mol (*L*-7 and *L*-1). The top 10 compounds are chosen for an additional in-depth investigation to identify SARS-CoV-2 major protease inhibitory drugs based on the docking score. We ran molecular dynamic simulations of *L*-1, *L*-7, and *L*-16 to test their stability in our biological systems after examining all the findings and comments. After comparing all of the outcomes to the FDA-approved antiviral drug remdesivir, the following compounds are suggested as improved SARS-CoV-2 inhibitory lead compounds: fumarostelline, strychnidin-10-one (*L*-1), 2,3-dimethoxy-brucin (*L*-7), and the alkaloid ND-305B (*L*-16). Based on the study of molecular docking, protein ligand interactions of docked complexes, and surface molecular electrostatic potentials, our proposed mechanism is as follows: the formation of the PHE294, ARG298, and GLN110 residues by our proposed lead compounds may limit the function of the viral protein *M*^pro^ (6M03) to combat SARS-CoV-2. On the other hand, suggested compounds can also be used for further in vitro and in vivo studies.

## Figures and Tables

**Table 1:** Ligand numbers, PubChem CID, and binding energy of 50 alkaloids.

S/N	L/N	CID	B.E
1	***L*-1**	**182165**	**−8.5**
2	*L*-2	102115610	−7.9
3	*L*-3	102115616	−7.8
4	*L*-4	102115614	−7.7
5	*L*-5	42608134	−7.6
6	*L*-6	102115592	−7.6
7	***L*-7**	**220520**	**−8.5**
8	*L*-8	102115609	−7.4
9	*L*-9	621853	−7.4
10	*L*-10	102115619	−7.4
11	*L*-11	536061	−7.3
12	*L*-12	5462444	−7.3
13	*L*-13	10154	−7.3
14	*L*-14	102115621	−7.2
15	*L*-15	101821325	−7.1
16	***L*-16**	**102115603**	**−8.2**
17	*L*-17	3034034	−7.1
18	***L*-18**	**102115620**	**−8.2**
19	*L*-19	969488	−7.1
20	*L*-20	6434971	−7.1
21	***L*-21**	**102115604**	**−8.1**
22	*L*-22	102115615	−7.1
23	*L*-23	21769952	−7.1
24	*L*-24	185716	−7.1
25	***L*-25**	**102115597**	**−8.0**
26	*L*-26	250873	−7.1
27	*L*-27	14081836	−7.0
28	*L*-28	73404	−7.0
29	*L*-29	101288388	−7.0
30	***L*-30**	**119204**	**−7.9**
31	*L*-31	102115590	−7
32	*L*-32	611742	−6.9
33	*L*-33	14488091	−6.9
34	*L*-34	5460437	−6.9
35	***L*-35**	**235224**	**−7.9**
36	*L*-36	6442501	−6.9
37	*L*-37	14589893	−6.8
38	*L*-38	5321926	−6.8
39	*L*-39	100978913	−6.8
40	***L*-40**	**101285909**	**−7.9**
41	*L*-41	92759	−6.7
42	*L*-42	21581112	−5.9
43	*L*-43	11008336	−5.8
44	*L*-44	121896	−5.7
45	*L*-45	442651	−5.6
46	*L*-46	3083764	−5.6
47	*L*-47	189721	−5.6
48	*L*-48	6430518	−5.6
49	*L*-49	333469	−5.5
50	***L*-50**	**11969631**	**−7.9**

B.E, binding energy.

**Table 2:** Top 10 docking scores of selected alkaloids compared with other approved antivirals for *M*^pro^ (6M03).

S/N	L/N	B.E
1	*L*-1	−8.5
2	*L*-7	−8.5
3	*L*-16	−8.2
4	*L*-18	−8.2
5	*L*-21	−8.1
6	*L*-25	−8.0
7	*L*-30	−7.9
8	*L*-35	−7.9
9	*L*-40	−7.9
10	*L*-50	−7.9
11	Remdesivir	−7.8
12	Ritonavir	−7.2
13	Lopinavir	−7.3
14	Oseltamivir	−6.1
15	Ribavirin	6.0
16	Remdesivir	−7.8

**Table 3:** Interactions of the protein (*M*^pro^) with the top ten alkaloids.

Serial No.	Compound	Hydrogen bond		Hydrophobic bond	
		Residues	Distance (Å)	Residues	Distance (Å)
1	*L*-1	THR111	3.10	PHE305	5.04
		ASP153	3.68	VAL303	3.71
				**PHE294**	3.27

2	*L*-7	**GLN110**	**2.03**	**ARG298**	5.10
		**PHE294**	3.43	VAL104	3.93
				ILE106	5.23

3	*L*-16	Absent	Absent	VAL303	4.70
				VAL297	5.30
				**ARG298**	4.31
				PHE305	5.18
				PHE8	5.87

4	*L*-18	ASP295	2.86	**ARG298**	4.30
				**PHE294**	4.44

5	*L*-21	ARG131	3.05	MET276	5.03
		LYS137	5.58		
		LEU287	2.00	LEU272	5.42
		TYR237	3.56		

6	*L*-25	THR 292	2.74	PHE305	5.20
		**GLN110**	2.25	VAL104	4.98
		ASN151	3.32	ASP295	4.99
		ASP153	3.55		

7	*L*-30	THR292	2.44	**PHE294**	2.44
		ASP153	3.78		

8	*L*-35	THR292	4.31	**PHE294**	5.93
		ASP153	**1.53**		

9	*L*-40	**GLN110**	**1.95**	**ARG298**	4.83
				**PHE294**	4.47
				**PHE294**	5.23

10	*L*-50	GLY143	2.47	MET49	4.49
		ASN142	2.17	CYS145	5.20
		HIS 41	2.15	MET165	5.12

11	Remdesivir	**PHE294**	2.92	ASN131	2.90
		ASP293	3.17	VAL208	3.78
		THR111	2.16	PRO292	2.92
		**GLN110**	2.94	VAL 308	4.52
				PHE305	5.13

**Table 4:** Molecular and pharmacokinetic properties of selected potential alkaloids.

L\N	MW (g/mol)	H. Ac	H. Do	Log Po/w	Log S	TPSA (Å^2^)	BBB (+ve/−ve)	AOT	CAR	BA	PPB	Drug likeliness (no. of violation)				
												*L*	*G*	*V*	*E*	*M*
*L*-1	369.4	7	2	0.00	−3.45	88.5	+	III	NC	0.55	1.00	0	0	0	0	0
*L*-7	394.5	5	0	3.00	−2.92	51.2	+	III	NC	0.55	0.71	0	0	0	0	0
*L*-16	305.4	3	2	2.39	−3.56	49.9	+	III	NC	0.55	1.04	0	0	0	0	0
*L*-18	354.4	5	2	0.00	−3.56	61.8	+	III	NC	0.55	0.94	0	0	0	0	0
*L*-21	363.4	5	2	0.00	−3.62	76.2	+	III	NC	0.55	1.19	0	0	0	0	0
*L*-25	366.4	5	1	1.59	−3.41	73.5	+	III	NC	0.55	0.90	0	0	0	0	0
*L*-30	279.3	3	0	3.08	−4.00	21.70	+	III	NC	0.55	0.91	0	0	0	0	0
*L*-35	279.3	3	0	3.08	−4.00	21.70	+	III	NC	0.55	0.91	0	0	0	0	0
*L*-40	327.3	5	0	3.32	−3.01	43.46	+	III	NC	0.55	0.91	0	0	0	0	0
*L*-50	351.3	7	2	2.93	−2.09	96.30	+	II	CA	0.55	0.66	0	0	0	0	0
D-1	602.6	13	4	−0.14	−3.47	204.0	+	III	NC	+	0.88	2	3	2	1	3
D-2	720.9	7	4	4.42	−3.88	148.12	−	III	NC	0.01	−	2	4	2	1	4
D-3	628.8	5	4	4.32	−4.81	272.27	−	III	NC	0.55	−	1	3	1	0	3
D-4	332.4	5	2	2.10	−2.50	169.93	−	III	NC	0.55	−	0	0	0	0	0
D-5	244.2	8	4	−3.01	−1.71	95.440	−	III	NC	0.55	−	0	1	1	1	0

*D*-1: remdesivir; *D*-2: ritonavir; *D*-3: lopinavir; *D*-4: oseltamivir; *D*-5: ribavirin; +: present; −: absent.

**Table 5:** ADMET properties of the selected 10 ligands compared with 05 standard antivirals.

L/N	Human intestinal absorption (%)	Caco-2permeability	P-glycoproteininhibitor	P-glycoproteinsubstrate	Renal organic cation transporter	CYP3A4 substrate	Total clearance	AMES toxicity
*L*-1	99.045	1.319	No	Yes	No	Yes	1.001	No
*L*-7	96.125	1.143	Yes	Yes	Yes	Yes	0.996	No
*L*-16	95.321	1.289	Yes	Yes	Yes	Yes	1.245	No
*L*-18	94.340	1.069	No	Yes	No	Yes	0.962	No
*L*-21	94.862	1.212	Yes	Yes	Yes	Yes	1.250	No
*L*-25	98.662	1.025	No	No	Yes	No	0.502	No
*L*-30	96.771	1.716	Yes	Yes	Yes	Yes	1.023	Yes
*L*-35	96.771	1.716	Yes	Yes	Yes	Yes	1.023	Yes
*L*-40	95.009	1.245	No	No	No	Yes	1.140	No
*L*-50	71.228	0.375	No	Yes	No	No	0.443	Yes
Remdesivir	71.109	0.635	Yes	Yes	—	—	—	—
Ritonavir	96.388	1.196	No	Yes	Yes	Yes	0.607	No
Lopinavir	65.607	0.063	Yes	Yes	—	—	—	—
Oseltamivir	96.125	1.143	Yes	Yes	Yes	No	0.996	No
Ribavirin	96.125	1.143	Yes	Yes	Yes	No	0.996	No

—: not available.

**Table 6:** Chemical reactivity of recommended lead compounds compared with remdesivir.

L/N	HOMO (eV)	LUMO (eV)	HOMO-LUMO gap (eV)	Chemical potential	Hardness	Softness	Electronegativity	Electrophilicity
*L*-1	−0.197	−0.068	0.129	−0.132	0.064	15.503	0.132	0.136
*L*-16	−0.196	−0.022	−0.109	0.109	0.087	11.494	0.109	0.068
Remdesivir	−0.222	−0.051	0.170	−0.136	0.136	0.085	−0.136	0.110

## Data Availability

The datasets used and analyzed during the current study are available from the corresponding authors on reasonable request.

## Supplementary Materials

Supplementary Materials Supplementary 1: Table S1: pass prediction analysis of 50 alkaloids with their antiviral activity (Pa > Pi). Supplementary 2: Table S2: protein ligand interaction and identification of binding residues (alkaloids).
