# Supplementary material for: A Computational Study on Selected Alkaloids as SARS-CoV-2 Inhibitors: PASS Prediction, Molecular Docking, ADMET Analysis, DFT, and Molecular Dynamics Simulations
Source: Biochem Res Int. 2023 May 3;2023:9975275. doi: 10.1155/2023/9975275 (PMC10171978; doi:10.1155/2023/9975275)
Supplement: Supplementary Materials — Supplementary 1: Table S1: pass prediction analysis of 50 alkaloids with their antiviral activity (Pa > Pi). Supplementary 2: Table S2: protein ligand interaction and identification of binding residues (alkaloids). [file 9975275.f1.docx]

**A Computational Study on Selected Alkaloids as SARS-CoV-2 Inhibitors: PASS Prediction, Molecular Docking, ADMET Analysis, DFT and Molecular Dynamics Simulations**

**s**

**Golam Mortuza^a,^  Md Abul Hasan Roni^b*^, Ajoy Kumer^c^, Suvro Biswas^d^, Md. Abu Saleh^d*^, Shirmin Islam^d^, Samia Sadaf^e^, Fahmida Akther^f^**

^a,b^Department of Pharmaceutical Sciences, North South University, Dhaka-1217,Bangladesh.

^b^Department of Science and Humanities, Bangladesh Army International University of Science and Technology, Cumilla-3500, Bangladesh.

^c^Department of Chemistry, European University of Bangladesh-EUB, Dhaka-1216, Bangladesh.

^d^Miocrobiology Laboratory, Department of Genetic Engineering and Biotechnology, University of Rajshahi, Rajshahi-6205, Bangladesh.

^e^Department of Genetic Engineering and Biotechnology, University of Chittagong, Chittagong-4331, Bangladesh

^f^Department of Pharmacy, University of Chittagong, Chittagong-4331, Bangladesh

**Correspondence to:** [**roni_chem@baiust.edu.bd**](mailto:roni_chem@baiust.edu.bd)

**saleh@ru.ac.bd**

**Table S1**: **Pass prediction analysis of 50 alkaloids with their antiviral activity (Pa>Pi).**

| S/N | L/N | CID | Pa | Pi | B.E | S/N | L/N | CID | Pa | Pi | B.E |
| --- | --- | --- | --- | --- | --- | --- | --- | --- | --- | --- | --- |
| 1 | L-1 | 182165 | 0.354 | 0.052 | -8.4 | 26 | L-26 | 250873 | 0.385 | 0.122 | -7.1 |
| 2 | L-2 | 102115610 | 0.361 | 0.142 | -7.9 | 27 | L-27 | 14081836 | 0.450 | 0.048 | -7 |
| 3 | L-3 | 102115616 | 0.416 | 0.075 | -7.8 | 28 | L-28 | 73404 | 0.385 | 0.122 | -7 |
| 4 | L-4 | 102115614 | 0.335 | 0.186 | -7.7 | 29 | L-29 | 101288388 | 0.384 | 0.110 | -7 |
| 5 | L-5 | 42608134 | 0.654 | 0.009 | -7.6 | 30 | L-30 | 119204 | 0.482 | 0.008 | -7.9 |
| 6 | L-6 | 102115592 | 0.388 | 0.120 | -7.6 | 31 | L-31 | 102115590 | 0.380 | 0.053 | -7 |
| 7 | L-7 | 220520 | 0.445 | 0.005 | -8.5 | 32 | L-32 | 611742 | 0.584 | 0.023 | -6.9 |
| 8 | L-8 | 102115609 | 0.408 | 0.083 | -7.4 | 33 | L-33 | 14488091 | 0.433 | 0.037 | -6.9 |
| 9 | L-9 | 621853 | 0.396 | 0.048 | -7.4 | 34 | L-34 | 5460437 | 0.432 | 0.037 | -6.9 |
| 10 | L-10 | 102115619 | 0.394 | 0.097 | -7.4 | 35 | L-35 | 235224 | 0.482 | 0.008 | -7.9 |
| 11 | L-11 | 536061 | 0.450 | 0.048 | -7.3 | 36 | L-36 | 6442501 | 0.673 | 0.003 | -6.9 |
| 12 | L-12 | 5462444 | 0.659 | 0.009 | -7.3 | 37 | L-37 | 14589893 | 0.405 | 0.086 | -6.8 |
| 13 | L-13 | 10154 | 0.357 | 0.050 | -7.3 | 38 | L-38 | 5321926 | 0.400 | 0.091 | -6.8 |
| 14 | L-14 | 102115621 | 0.410 | 0.080 | -7.2 | 39 | L-39 | 100978913 | 0.370 | 0.128 | -6.8 |
| 15 | L-15 | 101821325 | 0.584 | 0.023 | -7.1 | 40 | L-40 | 101285909 | 0.451 | 0.048 | -7.9 |
| 16 | L-16 | [102115603](https://pubchem.ncbi.nlm.nih.gov/compound/102115603) | 0.443 | 0.053 | -8.2 | 41 | L-41 | 92759 | 0.507 | 0.047 | -6.7 |
| 17 | L-17 | 3034034 | 0.435 | 0.036 | -7.1 | 42 | L-42 | 21581112 | 0.396 | 0.048 | -5.9 |
| 18 | L-18 | 102115620 | 0.334 | 0.188 | -8.2 | 43 | L-43 | 11008336 | 0.586 | 0.022 | -5.8 |
| 19 | L-19 | 969488 | 0.430 | 0.063 | -7.1 | 44 | L-44 | 121896 | 0.405 | 0.105 | -5.7 |
| 20 | L-20 | 6434971 | 0.790 | 0.001 | -7.1 | 45 | L-45 | 442651 | 0.586 | 0.022 | -5.6 |
| 21 | L-21 | 102115604 | 0.526 | 0.017 | -8.1 | 46 | L-46 | 3083764 | 0.470 | 0.010 | -5.6 |
| 22 | L-22 | 102115615 | 0.408 | 0.083 | -7.1 | 47 | L-47 | 189721 | 0.457 | 0.072 | -5.6 |
| 23 | L-23 | 21769952 | 0.399 | 0.047 | -7.1 | 48 | L-48 | 6430518 | 0.455 | 0.045 | -5.6 |
| 24 | L-24 | 185716 | 0.394 | 0.098 | -7.1 | 49 | L-49 | 333469 | 0.479 | 0.033 | -5.5 |
| 25 | L-25 | 102115597 | 0.390 | 0.016 | -8.0 | 50 | L-50 | 11969631 | 0.415 | 0.076 | -7.9 |

Pa: Probability of activity, Pi= Probability of inactivity, B.E: Binding Energy

**Table S2: Protein ligand interaction and identification of binding residues (Alkaloids).**

| **Ligand NO** | **Drug: PubChem CID** | **2D MODEL (PDB ID:** 6M03**)** |
| --- | --- | --- |
| L-1 | [182165](https://pubchem.ncbi.nlm.nih.gov/compound/182165) | 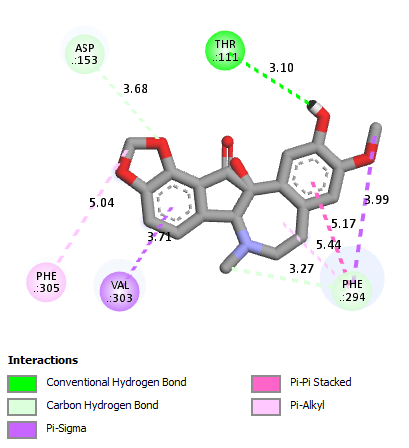  **Active side:**  **Hydrogen Bond: THR111(3.10)**  **Hydrophobic Bond**: ASP153(3.68), PHE305(5.04), VAL303(3.71), PHE294(3.27, 5.44, 5.17, 3.99) |
| L-7 | [220520](https://pubchem.ncbi.nlm.nih.gov/compound/220520) | 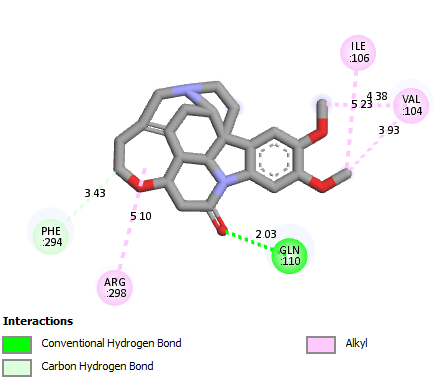  **Active side:**  **Hydrogen Bond: GLN110(2.03)**  **Hydrophobic Bond: *PHE294 (3.43), ARG298(5.10), VAL104(3.93,4.38), ILE106(5.23)*** |
| L-16 | 102115603 | 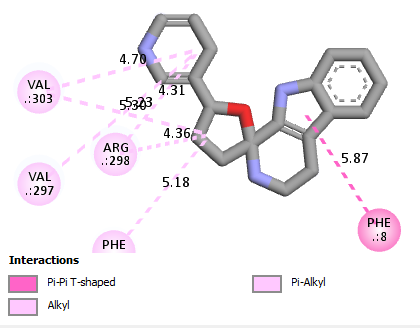  **Active side:**  **Hydrophobic : VAL303(4.70,5.23), VAL297(5.30), ARG298(4.31, 4.36), PHE305 (5.18), PHE8(5.87)** |
| L-18 | [102115620](https://pubchem.ncbi.nlm.nih.gov/compound/102115620) | 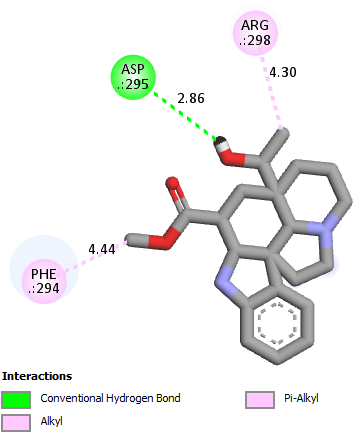  **Active side:**  **Hydrogen Bond**: ASP295(2.86),  **Hydrophobic Bond:**ARG298(4.30), PHE294(4.44). |
| L-21 | [102115604](https://pubchem.ncbi.nlm.nih.gov/compound/102115604) | 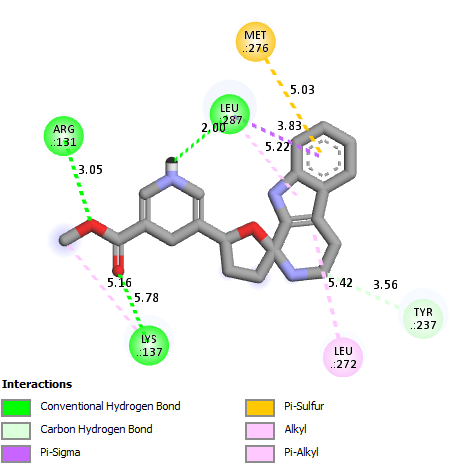  **Active side:**  **Hydrogen Bond**: ARG131(3.05), LYS137(5.58), LEU287(2.00), TYR237(3.56).  **Hydrophobic Bond:** MET276(5.03), LEU272(5.42) |
| L-25 | 102115597 | 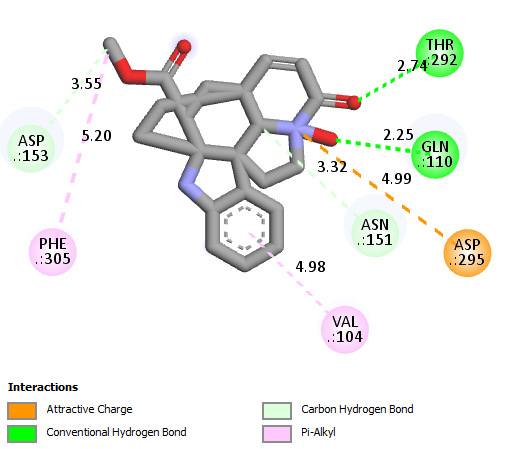  **Active side:**  **Hydrogen bond:** THR 292(2.74), GLN110(2.25), ASN151(3.32), ASP153(3.55).  **Hydrophobic Bond**: PHE305(5.20), VAL104(4.98), ASP295(4.99) |
| L-30 | 119204 | 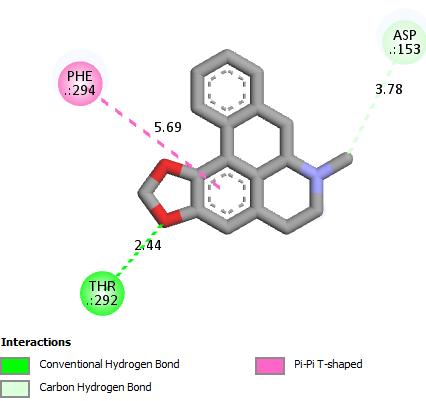  **Active side:**  **Hydrogen bond:** THR292(2.44), ASP153 (3.78)  **Hydrophobic Bond**: PHE294 (2.44) |
| L-35 | 235224 | 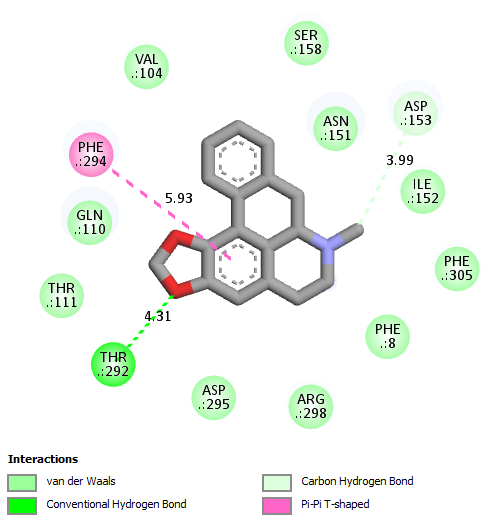  **Active side:**  **Hydrogen bond:** THR292(4.31), ASP153(153),  **Hydrophobic Bond**: PHE294(5.93) |
| L-40 | 101285909 | 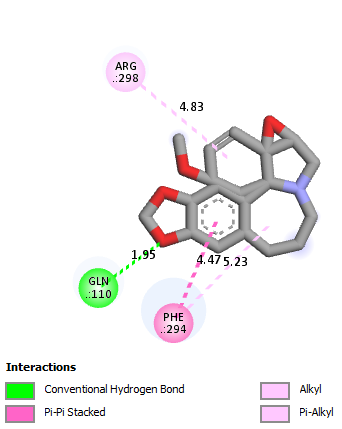  **Active side:**  **Hydrogen bond:** GLN110(1.95)  **Hydrophobic Bond**: ARG298(4.83), PHE294(4.47,5.23) |
| L-50 | 11969631 | 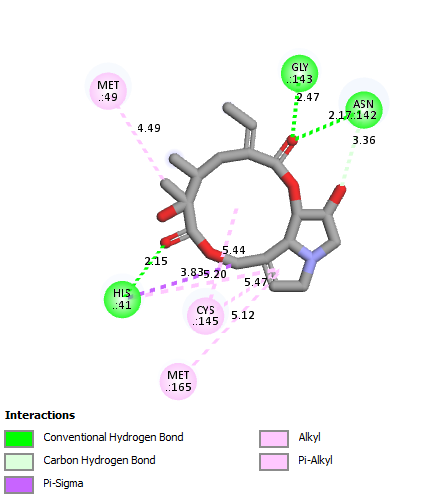  **Active side:**  **Hydrogen bond:** GLY143(2.47), ASN142(2.17- 3.36), HIS41(2.15)  **Hydrophobic Bond**: MET49(4.49), CYS145(5.20), MET165(5.12) |

**e.g., Lipinski’s “rule of five”: MW<500, LogP<5, hydrogen acceptor <10, and hydrogen donor <5) (Lipinski et al., 2001; Verheij, 2006) might cause problems later during the lead development stage (Verheij, 2006).**
